# A Nepalese bipolar case amidst risk and protective factors during COVID‐19 pandemic

**DOI:** 10.1002/ccr3.5523

**Published:** 2022-03-01

**Authors:** Dhana Ratna Shakya, Sandesh Raj Upadhaya

**Affiliations:** ^1^ 58594 Department of Psychiatry BP Koirala Institute of Health Sciences Dharan Nepal

**Keywords:** bipolar disorder, COVID‐19, protective factor, risk Factor, social support

## Abstract

Bipolar disorder is a chronic and recurring psychiatric illness. Emphasis on enhancing key protective factors like social support systems and promoting this to minimize risk factors like non‐compliance is one of the key strategies tied to enhance overall psychological, intellectual, and emotional health for symptoms remission and relapse prevention even during adverse situations like the COVID‐19 crisis. We intend here to point out implication of the dynamics of the protective and risk factors for bipolar mood through a 23‐year patient from eastern Nepal, which is largely harmonious in its multi‐ethnic, multilingual and multicultural social composition. This attribute of social cohesiveness and compassion is evident in this case report. When disturbed and ill, neighbors from native semi‐urban Nepalese society did support even in the times of crisis of COVID‐19 pandemic. The support of other people including neighbors is a key factor for the short‐term and long‐term management of bipolar mood.

## Graphical abstract

It is important in the management of bipolar case to enhance protective factors like social support systems and to minimize risk factors like non‐compliance for symptoms remission and relapse prevention even during the COVID‐19 Pandemic.

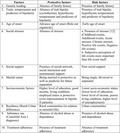

## 1 INTRODUCTION

Bipolar disorder is a severe and chronic illness with recurrent episodes of mania and depression. The overall lifetime prevalence of bipolar spectrum disorders, based on a large cross‐sectional survey, was 2.4%, with an account of 0.6% prevalence for bipolar type I and 0.4% for bipolar type II.^1^ The notion of risk and protective factors is one of the important parameters linked with predicting the outcome of bipolar disorder. These factors, of various forms as bio‐psycho‐social, have multidimensional operations starting from individual to family, school, neighborhood, and a broader societal level. The more chance of having the positive effects of protective factors that outweighs negative risk factors, the better is the prognosis. Major protective factors for a positive outcome in a bipolar patient include social support (from at least one caring adult), which is protective about a wide range of adversities.^2^

The Second wave of COVID‐19 was recorded in Nepal by April/May 2021, after the exponential rise of cases.^3^ We report a case of 23 years old man who was rescued by the neighbors after the exacerbation of the second episode of mania and brought to our center amid the COVID‐19 second wave.

## 2 CASE PRESENTATION

A 23‐year‐old unmarried Hindu male from Bishwakarma (ironsmith), a Dalit (oppressed) ethnicity,^4^ low middle socioeconomic status, and educated up to the third standard had been brought to our institute emergency department (E.D.) 3 years back for the sudden abnormal change in behavior.

The history of substance use was present in a problematic pattern in his father and three elder brothers. The mother passed away when the patient was 5 years of age. Father committed suicide by hanging after 6 months of the death of their mother under the influence of alcohol. The third elder brother with a history of multiple substance use (cannabis, benzodiazepine, opioids, and alcohol) was kept in prison for possession of an illegal amount of cannabis and involvement in a fight and robbery. He died during his stay in prison.

At the age of 13 years, the patient took his first puff of tobacco smoke under influence of his peer and the pattern of use was 2–3 sticks per day. Two days later of tobacco smoking, he started using marijuana out of curiosity and the current pattern was 1–2 sticks every 3–4 days. Three years later, he started using tablets of Nitrazepam 5 mg 2 tablets on and off that, as per the patient, gave him more energy while working as a conductor. Later, he used Spasmoproxyvon, Nitrazepam, and Opidol in combination, with 10 tablets per day, from his peer circle. If not available, he also used cough syrup (opioids), shared among friends. He would occasionally use locally distilled alcohol, the amount being 50 ml/day. The last use of all substances was 7 days before the presentation to our hospital.

After damaging a battery connecting to the wrong charging point and asking the workplace supervisor for money with an excessive desire to get married soon, he was fired from his job. He entered one of the neighbor's houses without permission and requested a young girl to get married to him. The patient started talking in a loud tone, the content of being a billionaire, having excessive money and power, and changing the topic frequently. He started playing television in a loud volume and cleaning the television and furniture at night time. He started overdemanding for food items asking for tea without finishing the current meal which was served to the patient. Despite a decreased number of hours of sleep, he appeared fresh and energetic in the morning roaming in his place. He was brought forcefully by his family members to an emergency. Initially, he was admitted with a diagnosis of substance‐induced psychotic disorder with harmful use of nicotine, cannabis, benzodiazepine, opioids, and alcohol. The Clinical Opiate Withdrawal Scale (COWS) score was 3. Blood investigation parameters were within normal limits. Young Mania Rating Scale (YMRS) was 36/60 at the time of admission. However, during the ward course of 43 days, he had a persistent mood elevation with the delusion of grandiosity and lack of insight. The diagnosis was revised to mania with psychotic symptoms. He gradually recovered after Olanzapine was optimized up to 30 mg. Lithium 900 mg was added as an augmenting agent for the predominant euphoric picture. Injectable Haloperidol 10 mg and Promethazine 50 mg were kept initially for behavioral control. At the time of discharge, the YMRS was 2/60. He was non‐compliant 2 months after the discharge and was in the same pattern of substance use until the current illness started.

After the death of his parents, the eldest brother who was the primary caretaker continued using alcohol in the problematic pattern. The patient was brought up in a community belonging to different ethnic castes who were concerned about the patient's illness. Three years later, the patient was brought to this hospital by his neighbors who belong to different ethnicity castes (Rai and Tamang, i.e., Janjati and Shrestha, i.e., Newar)^4^ due to abnormal behavior at home as his eldest brother did not show any concern toward his current behavior. He got married to a girl during the current illness period but separated 5 days later when she became aware of the illness. Like the previous episode, he had a similar elevated mood picture with the delusion of grandiosity and was difficult to control at home over 1 month period. Last use of alcohol and marijuana was 8 days prior to presentation to our hospital. So, he was admitted to our institution again on June 25, 2021, with a diagnosis of bipolar affective disorder, current episode manic with psychotic symptoms with cannabis and alcohol use. During the hospital stay, initial financial support was provided by three neighbors with frequent visits to our ward. The YMRS was 30/60 at the time of admission. Olanzapine 30 mg, Divalproex 1000 mg, and Lorazepam up to 6 mg were required during the ward course. Injectable Haloperidol and Promethazine were needed for behavioral control during the first 2 weeks as the patient's symptoms aggravated during ward stay. He had multiple attempts of trying to escape from our institution. Gradual recovery occurred after 17 days and the YMRS was 7 on day 23. The patient developed fever and running nose before discharge; however, PCR for COVID‐19 was negative. After fever decreased, the patient was discharged from our ward, with identification and psycho‐education to the caretakers, including the neighbors, to ensure compliance and follow‐up. The patient was doing well in the follow‐up period of 2 months.

## 3 DISCUSSION

Early parental loss is one of the specific life events linked with risk for mood/bipolar disorder, which was present in our case.^1^ Family history of substance use, history of suicide in father, lack of familial support, multiple substance use by patients including alcohol, and non‐compliance are the major risk factor for relapse in our patient.^5^ Cannabis use has been reviewed to be associated with increased incidence of bipolar disorder^6^ and our case was using cannabis every 3–4 days. There is a complex relationship between marriage and mood disorders. Being married is protective. However, the illness period can be distressing for spouses and can result in separation which can itself be a risk factor for future relapses.^5^ The patient support system should be strong; neither over‐involved nor withdrawing from the patient. Rapidly deteriorating symptoms and lack of a usual support system are some of the indications of hospitalization in bipolar disorder.^7^ This case needed hospitalization in the second episode even during COVID‐19 pandemic time to prevent further complications as the patient's condition was deteriorating. The patient's familial support system was broken and neighbors actively took a role in the management of current illness. Social support plays a protective role in bipolar disorder. One important parameter in prognosis is a decrement in the future relapse by improving medication adherence.^5^, ^8^ Consideration of non‐bipolar patients with improved cardiovascular and immune function by social support gives an indirect benefit of improved physical condition in bipolar patients.^8^ This is particularly important after discharge as continued support would help in decreasing future relapses.

The Eastern part of Nepal, like other parts, is largely harmonious in its multi‐ethnic, multilingual, and multicultural social composition despite traces of the caste system.^4^ In developing countries like Nepal, attitude toward mental illness is particularly based on religious and magical beliefs.^9^ Integration of cultural and ethnic values at the community and local level is essential to tackle the negative cycle of social determinants like poverty, low socioeconomic status, early childhood adverse experience, unemployment, stigma, and poor access to health services, which play a contributing role in causation, severity, course, and outcome of chronic mental illness like bipolar disorder.^5^, ^10^ This case report gives an exemplary idea that the supportive role of neighbors who belong to native semi‐urban Nepalese society can be of great importance in all levels of management of mental illness including bipolar mood. Support of other people including neighbors helps the patient receive acute treatment, attain symptom remission, and achieve overall physical, intellectual, and emotional health; even and more so during the COVID‐19 crisis as responsible citizens.^11^

It would be pertinent to review the following (Table 1) to understand some of the major risk factors and protective factors which might play varying roles for predisposition, precipitation, or perpetuation and relapse, that is, determining the overall fate of the course of bipolar disorder.^5^

**TABLE 1:** Protective and risk factors of bipolar mood

Factors	Protective factors	Risk factors
1. Genetic loading	Asbsence of family history	Presence of family history
2. Traits, temperament, and predictors of bipolarity	Absence of trait bipolar (cyclothymia), hyperthymic temperament, and predictors of bipolarity	Presence of trait bipolar, hyperthymic temperament and predictor of bipolarity
3. Age of onset	Advance age of onset (Rule out organicity)	Early age of onset
4. Social stressor	Absence of stressor	Pressence of stressor^12^ (Childhood events, Adulthood events, Acute stressor, Chronic stressor, Positive life events, Negative life events) Subjective perception of life events more important than life event itself
5. Social support	Presence of social network, social interaction, and instrumental support	Poor social support
6. Marital status	Being married is protective as well as predictor for future separation	Being single, divorced, or separated
7. Socioeconomic factors	Higher level of education, good income, living condition, employed status is protective (overrepresentation in bipolar II patients)	Lower socioeconomic status (lower level of education, lower level of income, poorer living condition, higher level of unemployment)
8. Residence (rural‐urban difference)	Rural communities (in relation to stressful life)	Urban communities
9. Substance use (comorbid axis I diagnosis)	Absence of alcohol abuse or dependence	Presence of alcohol abuse and dependence
10. Treatment adherence	Presence of treatment adherence	Absence of treatment adherence

## 4 CONCLUSIONS

This case report entails highlighting some of the illustrated protective and risk factors of bipolar mood through a 23‐year‐old man from eastern Nepal who became ill the second time during the second wave of COVID‐19. Even during adverse situations like this, harmonious relationships displayed by the neighbors in the community can play a role in the management of this recurring and chronic illness; such favorable social support is associated with a potentially good outcome even in bipolar illness. Considering the social context during admission and targeting the approaches to enhance the neighborhood and social support is one of the key strategies tied to improve patient outcomes.

## CONFLICT OF INTEREST

The authors state that there is no conflict of interest.

## AUTHOR CONTRIBUTIONS

DRS involved in conception, design, collection of data, and drafted and edited the manuscript. SRU collected the data, drafted the manuscript. DRS and SRU published the final approval of the version.

## ETHICAL APPROVAL

Ethics approval was not required.

## CONSENT

Written informed consent was obtained from the patient to publish this report in accordance with the journal's patient consent policy.

## Data Availability

The authors confirm that the data supporting the findings of this study are available within the article.
